# Cell-cycle genes in the mussel *Mytilus galloprovincialis* are cued by aerial heat stress in a fluctuating tidal environment

**DOI:** 10.1007/s00360-026-01691-y

**Published:** 2026-09-09

**Authors:** Helen C. Hong, Edwin A. Solares, Michael M. Abdelsayed, Kwasi M. Connor

**Affiliations:** 1 Department of Ecology and Evolutionary Biology, University of California, Irvine, CA 92697 USA; 2 Halıcıoğlu Data Science Institute, University of California, San Diego, CA 92093 USA; 3 Department of Biology, California State University, Northridge, CA 91330 USA

**Keywords:** Marine mussels, Cell cycle, Cyclin, Heat-stress, Hypoxia, Tidal cycle, RNA thermometers

## Abstract

*Mytilus galloprovincialis* is a sessile, facultative anaerobic mussel inhabiting intertidal zones, where it experiences daily fluctuations in oxygen, temperature, and food availability. These environmental fluctuations require coordinated shifts in metabolism and cellular processes such as the cell cycle. As growth in mussels is environmentally driven, understanding how external perturbations modulate cell-cycle regulation is essential. Despite conserved cell-cycle machinery across eukaryotes, its dynamics in facultative anaerobic marine invertebrates remain largely unexplored. Mussels switch between aerobic (high-tide submergence) and anaerobic (low-tide emergence) respiration over tidal fluctuations. We hypothesized that cell-cycle gene expression (cyclins) is synchronized with tidal cues, peaking during anaerobic metabolism (low tide) in control-acclimated mussels to avoid DNA damage from oxidative stress during aerobic metabolism. Alternatively, peak expression would occur during submergence after experiencing acute heat stress in air to avoid higher risk of DNA damage by aerial heat compared to damage by oxidative stress in cool water. We also predicted higher cyclin transcript abundance in heat-stressed mussels compared to control mussels. To test these inferences, we exposed mussels to simulated tidal conditions and measured gene expression of cyclins and stress markers. We performed an RNA/DNA ratio assay as a proxy for short-term growth. As predicted, acute heat-stressed mussels exhibited significantly elevated cyclin transcript abundance compared to control mussels. Unexpectedly, *cyclin D* and *E* transcripts were upregulated during low tide in the heat-stressed mussels, while peak *cyclin B* expression was delayed until post-stress resubmergence. Furthermore, we did not observe significant changes in RNA/DNA ratio across the treatments. Our findings demonstrate that external environmental cues can modulate the timing and magnitude of cell-cycle gene expression in *M. galloprovincialis*, advancing our understanding of growth regulation in facultative anaerobes. We also assessed the potential of cyclin gene expression regulation through RNA secondary structure (RNA thermometer).

**Supplementary Information:**

The online version contains supplementary material available at https://doi.org/10.1007/s00360-026-01691-y.

## Introduction

The marine mussel, *Mytilus galloprovincialis*, is a facultative anaerobic bivalve that inhabits intertidal zones worldwide. As a sessile species, *M. galloprovincialis* must cope with daily fluctuations of tide level and wave exposure which drives variability in salinity, oxygen, temperature, nutrient availability, and predation (Horn et al. 2021). *Mytilus* species are ecosystem engineers; aggregations of mussels provide substrate, protection, and food for a variety of organisms, and filter particulates from the water column. Adapted to fluctuating environmental conditions within bays and estuaries, it is an ideal model organism to advance our knowledge of the physiological underpinnings of facultative anaerobiosis.

During high tide, mussels within the genus *Mytilus* are submerged in water, open their shell valves, feed on suspended algae and zooplankton while simultaneously consuming oxygen. After the tide recedes, mussels are exposed to air and close their valves to avoid desiccation. The oxygen within their shell cavities is quickly consumed, forcing a metabolic switch from aerobic to anaerobic respiration (De Zwann 1976; Connor and Gracey 2012). As ectotherms, a mussel’s body temperature reflects its external environment (Helmuth 1998). Air temperature can often reach levels higher than surrounding water that can lead to alterations in physiology, cell damage, or death (Helmuth and Hofmann 2001; Gracey et al. 2008; Schneider et al. 2010, Logan et al. 2012, Miller and Dowd 2019; Lin et al. 2025). Therefore, mussels residing within the intertidal zone face various levels of periodic heat stress and functional hypoxia simultaneously during low tide and routinely activate heat shock response systems (HSR) which require high levels of ATP (Lindquist and Craig 1988; Gracey et al. 2008). Furthermore, *Mytilus* displays indeterminate growth, thereby heavily reliant upon environmental conditions rather than genetic factors (Sebens 1987). Mussels established high on the shore are subjected to longer periods of air-exposure, consume less food, and endure more heat stress than mussels low-shore. As a result, they display relatively lower somatic and reproductive growth rates (Petes, Menge et al. 2007, Schneider et al. 2010, Connor and Robles 2015). Growth and size influence key life-history traits, including competition, survival and fecundity, highlighting the importance of studying growth and size under fluctuating environments (Paine 1976; Bayne et al. 1983; Robles et al. 1990; Xavier et al. 2007).

**Fig. 1:**
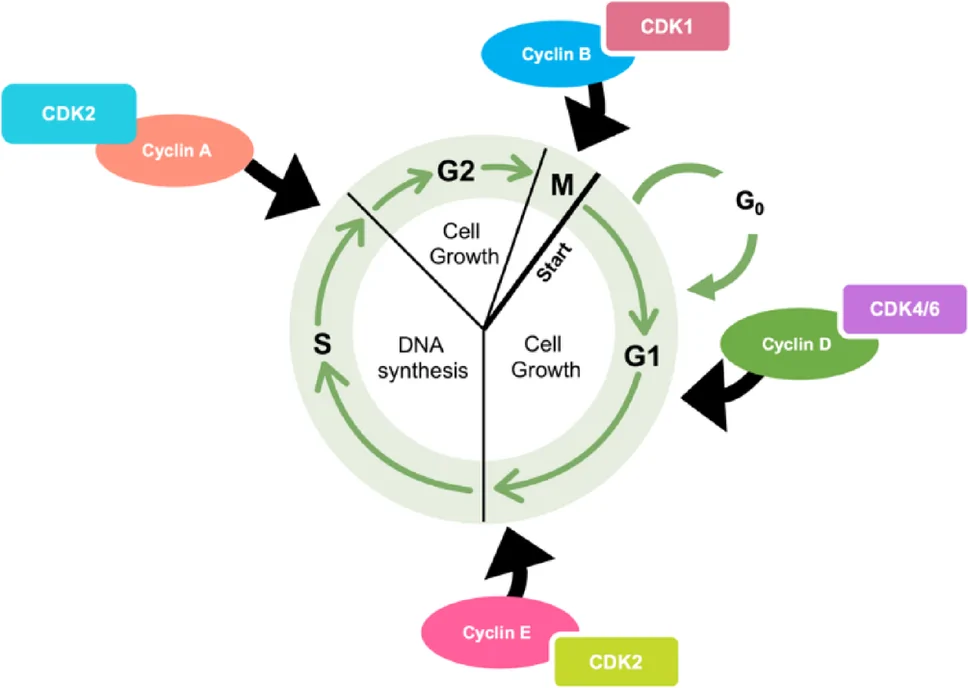
Cell-cycle phases and the specific cyclin/CDK complexes corresponding to each phase. The typical cell-cycle progresses in consecutive order, starting at G_1_ (growth), S (DNA synthesis), G_2_ (growth) and to M (mitosis) phase. The cell can enter G_0_ if the conditions are unfavorable for progression. During G_0_ stage, cells are considered quiescent/arrested in a non-proliferating state.

Eukaryotic growth is a function of cell division—a complex set of molecular processes integrated with the cell cycle. The cell cycle underlies somatic growth, repair, development, and differentiation of cells (Jakoby and Schnittger 2004; Zhang and Wang 2019). The well-studied mammalian cell cycle is a fine-tuned sequence of cellular events that progress in an orderly manner containing four main distinct phases: G_1_ (gap/growth), S (DNA synthesis), G_2_ (further growth) and M (mitosis) (Fig. 1). Its progression is driven by the activation and subsequent inactivation of two key regulatory moleculesࣧ —cyclins and cyclin-dependent kinases (CDKs) which form complexes that phosphorylate downstream targets. While CDKs are constitutively expressed throughout the cell cycle, cyclins are synthesized and degraded according to cell-cycle phases. A phase cannot commence without the completion of the previous phase (Yang 2018). The core cell cycle biochemical machinery such as cyclins, CDKs and other regulator proteins, are highly conserved across species (Menges et al. 2005; Cross et al. 2011). However, the cell cycle in alternative organisms may deviate from previously described mammalian systems. For instance, the yeast cell cycle has unique cellular mechanisms that lack certain regulator proteins conserved amongst other eukaryotes (Cross et al. 2011; Bähler 2005). In mussels, the prominent impact of environmental fluctuations on energy availability for indeterminate growth raises the question of whether the cell-cycle architecture is directly influenced by the tidal cycle or temperature. While fish are often used to study the influence of marine environments on the cell cycle amongst animals, few studies have investigated its processes in facultative anaerobic marine bivalves (Podrabsky and Culpepper 2012; Siefert et al. 2015).

There is an energetic trade-off between somatic growth and maintenance costs (Na^+^K^+^ pumps, HSR, protein turnover) in mussels. Hence, the cell cycle must be strictly regulated to maintain energy efficiency and balance. Furthermore, uncorrected mistakes in cell-cycle progression can lead to cell death or uncontrollable growth. Hence, mussels potentially coordinate cell cycle and proliferation (elevated cell-cycle rate) with the tidal environment as it temporally regulates biological factors such as nutrient acquisition, oxygen consumption, and body temperature which in turn may directly (e.g., DNA damage) or indirectly (e.g., low energy storage level) modulate cell-cycle progression. Within the framework of comparative physiological analyses, cell-cycle dynamics of the model metazoan facultative anaerobe *Caenorhabditis elegans* have previously been observed in fluctuating stressful environments. However, these studies are often limited to growth during development and typically under a single environmental stressor (Shen et al. 2005; Kitazume et al. 2018; Fawcett et al. 2015), limiting our knowledge of growth in adult facultative anaerobic animals undergoing environmental stress.

Mussels of the genus *Mytilus* possess mammalian cell cycle genetic orthologues, highlighting its conservation over evolutionary timescales (Gracey et al. 2008; Murgarella et al. 2016, Paggeot et al. 2022). These include cyclins, CDKs, and CDK inhibitors. Additionally, mammalian studies revealed that tumor suppressor p53, regulates CDK inhibitor p21, and therefore, plays an important role in arresting cell-cycle progression at the pre-mitotic phase—halting the transition from G_2_ to M phase; reviewed in Vermeulen (2003a). In this context, a field study of *M. californianus* analyzed its transcriptome over tidal microhabitats, revealing robust fluctuations in cell division genes (Gracey et al. 2008). Genes related to cell division oscillated in mussels acclimatized to a thermally stressful high-shore location and a less-stressful low-shore environment. However, mussels high on the shore exhibited greater transcription of a cluster of cell division genes than mussels low shore, suggesting that the environment modulates the cell cycle in *Mytilus.* The relatively elevated expression of cell division genes in high-shore mussels may have been driven by tide or temperature. However, the stochastic nature of the intertidal zone prevented direct associations with these variables (i.e., a lack of controls).

The discovery of higher expression levels of cell-cycle genes in thermally challenged high-shore mussels compared to those situated low shore by Gracey et al. (2008) is intriguing considering that they grow slower than low-shore populations (Connor and Robles 2015). Hence, these analyses are suggestive of preparatory gene expression (i.e., elevated RNA abundance but delayed translation) to potentially mitigate environmental-stress related cell-turnover processes, as opposed to facilitating positive growth of tissue and shell. Indeed, differential environmental variables between microsites likely played a role in the observed alternate expression patterns between these two populations. However, laboratory settings may help isolate environmental variables driving these patterns. For example, they can allow for analyzing the timing differences in peak expression between stressed and non-stressed mussels in addition to differences in levels of expression. Remarkably, a recent study computationally modelled how environmental perturbations can modify the dynamics of cell-cycle components and progression in yeast (Adler et al. 2022), supporting our argument that intertidal environmental cues (tide or tide+heat) can modulate cell-cycle progression in marine mussels.

The logic of temporal partitioning of cellular processes has been defined through previous studies of yeastࣧ —a model facultative anaerobic eukaryote (Nielsen 2019). Importantly, yeast can be induced to display aerobic/anaerobic ultradian cycles similar to tidally driven metabolic cycles in mussels (Tu et al. 2005). Transcriptomic research of these cycles revealed that peak expression of cell-division genes occurred during the repetitive anaerobic periods. An explanation for this observation suggests that cell division in yeast occurs during periods of anaerobic metabolism to avoid damage to DNA that may occur during the formation of free radicals when respiring oxygen (i.e., oxidative stress). Therefore, the overarching goal of our study is to investigate how acute exposure to moderate environmental fluctuations driving normoxic/hypoxic cycles in mussels restrict the peak gene expression of cell-cycle genes to specific periods of the tidal cycle.

**Fig. 2:**
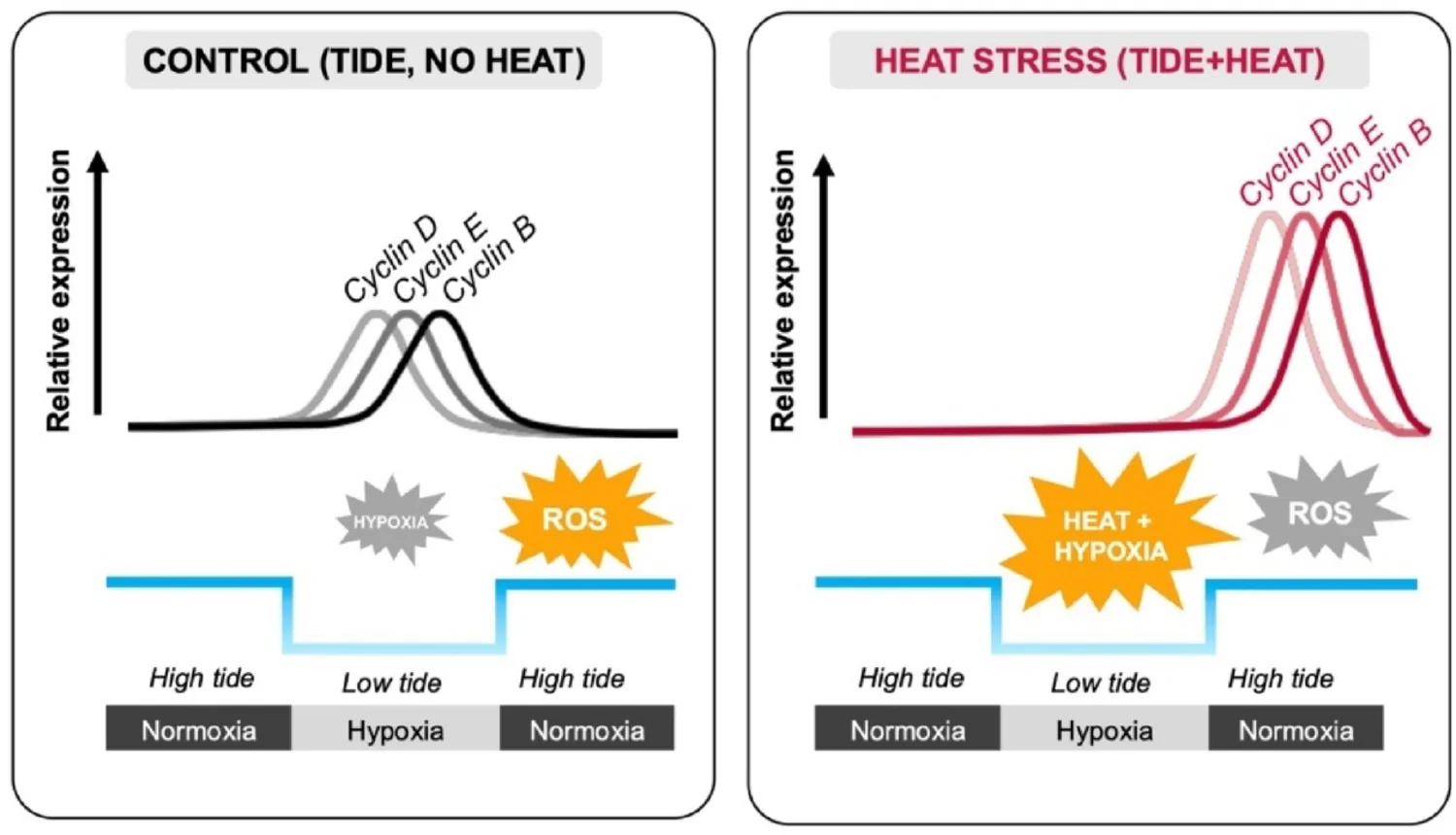
Predictions for cyclin expression throughout the tidal cycle for control (tide, no heat) and heat -stressed group (tide+heat). In the control group, we predicted that cyclin cascade (*cyclin D*, *E*, followed by *B*) would occur during low-tide functional hypoxia. During aerial heat-stress, we predicted that the cyclin cascade would be delayed to high-tide resubmergence period. Furthermore, we predicted that cyclin expression level would be higher in the heat-stressed group compared to control —peaks of curves are higher in heat stress group compared to the control group.

Using the logic of temporal partitioning in yeast, we predicted that under hypoxia/normoxia tidal cycles (control mussels at 15 °C), the cell-cycle cascade would begin with elevated *cyclin D* (*CCND2*) expression (regulatory marker of G1 phase) during functional-hypoxia (low tide), followed by *cyclin E* (*CCNE1*) gene expression (regulatory marker of transition from G1 to S phase) (Fig. 2). Lastly, the cell-cycle cascade would end with peak expression of *cyclin B* (*CCNB1*) with all cyclin peaks occurring during low-tide functional hypoxia to avoid oxidative stress during re-oxygenation of tissues that occurs during resubmergence (Fig. 2). Support for this inference derives from an observation of bursts of reactive oxygen species (ROS) due to increased respiration rates (i.e., oxygen debt) and lipid peroxidation following resubmergence in *M. edulis* (Rivera-Ingraham et al. 2013).

Peak gene expression shortly after environmental change is termed direct expression (direct impulse response)—adapted from Yosef and Regev (2011). In contrast to direct expression, gene expression that occurs considerably later is labeled delayed-expression (delayed impulse response)—adapted from the signal-sensitive delay concept (Yosef and Rege 2011). In this context, we hypothesized that mussels subjected to an acute (aerial) heat shock during low tide (functional hypoxia and heat stress) would delay the cyclin cascade. Peak expression of these genes would occur during resubmergence (high tide) to avoid thermally induced DNA damage while mitigating oxidative stress simultaneously (Fig. 2). Lastly, we predicted higher transcript abundance of cyclin genes in heat-stressed mussels than control individuals, in agreement with previous observations of differential gene expression patterns between high-shore thermally acclimatized mussels and those residing in a cooler low-shore microhabitat (Fig. 2) (Gracey et al. 2008). In an effort to characterize the environmental influence on cyclin regulation, we measured the RNA abundance of tumor suppressor protein p53 (*TP53*), which is a marker of cell-cycle arrest. Protein abundance can be controlled by mRNA translation rate, which in turn is thermally sensitive through RNA thermometers. RNA thermometers are transcripts that possess internal structures that fold RNA in a manner that does not allow for translation unless a certain temperature is met, typically through altering ribosome acessibility. Using secondary structure predictions, we analyzed the potential of thermally sensitive cell-cycle regulation by analyzing the potential of cyclins to be RNA thermometers. This study represents a novel approach to understanding cell-cycle regulation in mollusks.

## Methods

### Animals and acclimation

*Mytilus galloprovincialis* mussels averaging 69.9 mm (length), 35.0 mm (width) and 38.7 g (weight), age 6–8 months were obtained commercially from Carlsbad Aquafarm (Carlsbad, CA) (*N* = 60). Mussels were of similar age/size, and developed in the same environment. Somatic growth follows the Von Bertalanffy growth model characterizing rapid growth in young mussels. Mussels were transferred to the University of California, Irvine and held within a circulating aerated tank at 15 °C, which is near optimal temperature for *M*. *galloprovincialis* (Fearman and Moltschaniwskyj 2010). Mussels were allowed to purate for 48 h in constant light conditions to prevent entrainment to light-dark cycles (i.e., circadian rhythm) (Fearman and Moltschaniwskyj 2010; Connor and Gracey 2011).

**Fig. 3:**
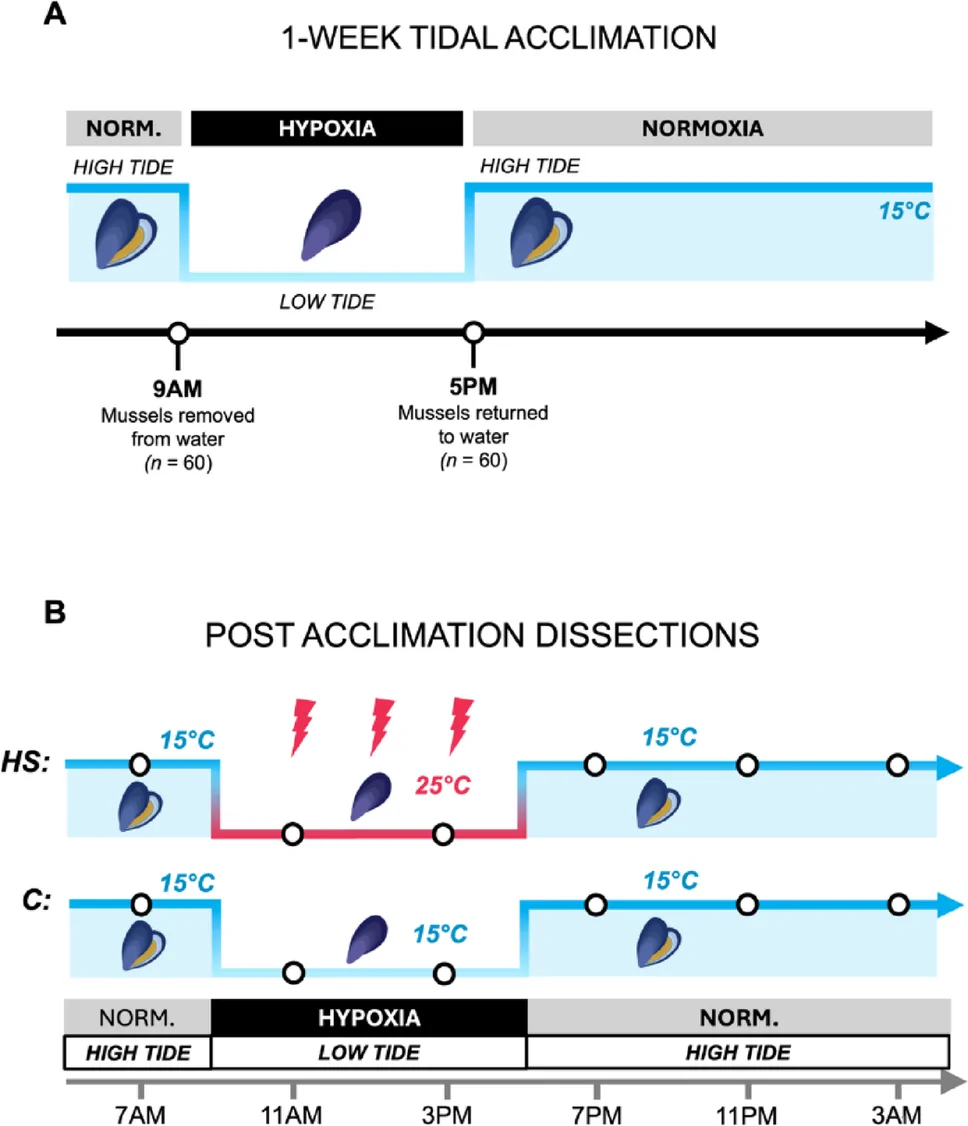
**A** One-week tidal acclimation scheme at 15 °C (*N* = 60). Tidal cycles were manually stimulated by removing mussels from the water at 9AM and return at 5PM. The aerial temperature was kept constant at 15 °C. Mussels were fully submerged during high tide and exposed to normal oxygen conditions (normoxia) in the form of dissolved oxygen in the water. During low tide, mussels were fully emerged from the water which induced shell-valve closure. Mussels were fed algae for two-hours after high tide (5-7PM). **B** Dissection schematic of *M. galloprovincialis* (*N* = 60). Each sampling time point (6 total) is indicated with open circles and corresponding open lines directing the time of dissection (4-h intervals, *N* = 5 mussels per experimental group at each timepoint). The heat-stressed group was acutely exposed to 25 °C during low tide, meanwhile the control group remained at 15 °C throughout the entire dissection. Light condition remained constant throughout acclimation and dissection (24 h light).

Mussels were acclimated to a laboratory simulated tidal cycle regime in two 15-gallon tanks with seawater (60:40 artificial: gravel filtered seawater) at 15 °C for one week (Fig. 3A). Low tide was simulated manually by temporarily removing mussels from the water for 8 h per day (9AM to 5PM). After low tide, mussels were returned to their tanks and fed live algae containing a mixture of *Isochrysis*, *Nannochloropsis*, *Tetraselmis*, and *Porphyridium* in equal parts (Filter Feeder Formula; Algae Research and Supply; Carlsbad, CA) with a final concentration of ~ 1,000 cells/mL—reference Maire et al. (2007) for associated filtration activity in *M. galloprovincialis*. Filter pumps were turned on after a 2-h feeding period to remove any remaining algae from the water, potentially synchronizing digestive activities and other cellular processes.

### Acute heat shock experiment and dissections

Following the one-week acclimation, mussels were sacrificed to collect gill tissue at 4-h intervals, with a total of six collection time points (Fig. 3B). Dissections from both tanks began 2 h prior to low tide (7AM) and the last dissection occurred 10 h after low tide (3 AM). At the beginning of low tide (9AM), a subset of mussels (*N* = 30) was removed from the water from the control tank and remained outside of the water at acclimation temperature (15 °C). Mussels from the treatment group (*N* = 30) were acutely heat stressed at 25 °C (aerial temperature) in an incubator. All dissected gill tissue was stored at −80 °C to preserve RNA for later use in gene expression and RNA/DNA ratio analyses. A total of five mussels were sacrificed at each time point from each group. No mortality was observed during the experiments.

### Total RNA extraction and RT-qPCR

To examine the gene expression in mussel samples, we extracted total RNA by lysing the frozen gill tissue using a bead mill homogenizer (TissueLyser II, QIAGEN) in TRIzol (Invitrogen Life Sciences). RNA concentration (ng/µL) and purity (260/280 ratio) were assessed using a Biotek Take3 spectrophotometer to confirm that the integrity of the isolated RNA was appropriate for downstream qPCR analysis. The isolated RNA for each sample had a 260/280 ratio that fell within the range of ~ 2.0–2.1, indicating high purity and negligible protein, phenol, or other contaminants.

Each RNA sample was diluted to 1000 ng/µL and used to synthesize cDNA (iScript cDNA Kit, Bio-Rad). The resulting cDNA product was used to analyze gene expression in a qPCR assay (CFX thermocycle, Bio-Rad). *Tubulin-alpha* (*TBA1A*) was used as the reference gene to normalize the expression of the target genes. Nucleotide sequence accessions were retrieved by performing BLAST (BLASTn). *Mytilus galloprovincialis* transcriptome shotgun assembly sequences were compared to *M. californianus* expressed sequence tags (ESTs) from Gracey et al. (2008). The *cyclin E* sequence was identified by aligning the *M. galloprovincialis* sequence with the *Crassostrea virginica cyclin E* target sequence from Li et al. (2022). Primers for oxidative stress-related genes were obtained from previously published studies (Table S1).

To confirm that mussels were properly heat stressed, we assessed the stress-response by measuring gene expression of inducible heat shock protein-70 (*HSPA12A*), anti-apoptotic (*BIRC4* and *BIRC7*), tumor suppressor protein p53 (*TP53*), and oxidative stress markers (*MnSOD*,* GST* and *CAT*). Heat shock proteins (HSPs) are chaperones that help refold denatured proteins from heat exposure. Anti-apoptotic genes encode proteins that have the potential to halt apoptosis to ensure cell survival.

### qPCR data preparation and relative gene expression calculation

Cq scores obtained from the qPCR assays were bootstrapped and randomized within each timepoint (25 permutations) in accordance with normalizing techniques in qPCR data analysis. Bootstrapping and randomization techniques correct for non-normal distribution outcomes formed by exponential components of relative expression calculations (Pfaffl et al. 2002; Clèries, 2012). The randomized permutations of the Cq scores of the target genes were matched with randomized Cq scores of the reference gene (tubulin-alpha) for each group individually. The number of permutations used allowed each pair to match at least twice within a group. The relative expression was subsequently calculated using the formula (Eq. 1):

1 $$\mathrm{Relative} {\mkern 1mu} \,\mathrm{expression} = 2^{{\left( {cq_{{_{{reference{\kern 1pt} gene}} }} - cq_{{_{{target{\kern 1pt} gene}} }} } \right)}}$$

### RNA/DNA ratio assay

Frozen gill tissue was cut into 5 mg pieces on dry ice. The extraction protocol was modified from the Nucleospin Triprep kit (Macherey-Nagel). DNA and RNA were extracted from the same tissue piece to maintain the consistency of the ratio. The purity (260/280 ratio) and concentration (ng/µL) of both nucleic acids for each sample were measured using a BioTek Take3 spectrophotometer. The RNA and DNA concentrations were subsequently used to calculate the RNA/DNA ratio.

### Statistical analysis

One-way ANOVA and post hoc Fisher’s least significant difference (LSD) tests were performed to statistically compare relative expression means of control and heat-stressed groups at each time point. Pairwise comparisons were performed by false discovery rate (FDR) adjustment using the Benjamini–Hochberg (BH) method. Significance was set to *p* < 0.05. RNA/DNA ratio comparisons were performed using a one-way ANOVA with a Tukey post hoc test. SPSS v29.0.1.0 was used to perform statistical tests.

### RNA secondary structure predictions

To determine secondary structure of 5’ UTRs of cyclin RNA, we verified their sequences by aligning *M. galloprovincialis* shotgun sequences (Table S1) to the *M. galloprovincialis* and *M. californianus* genomes along with previously annotated mRNA sequences. For *M. californianus* we aligned the same *cyclin B* Expressed Sequence Tag revealed differentially expressed in Gracey et al. (2008) to the *M. californianus* genome. Analyses were performed using *M. galloprovincialis* genome assembly xbMytGall1.hap1.1 (RefSeq accession GCF_965363235.1/BioProject PRJEB90278) generated by the Darwin Tree of Life Project, and the *M. californianus* genome assembly xbMytCali1.0.p (RefSeq accession GCF_021869535.1/BioProject PRJNA796333) (Paggeot et al. 2022) (Fig. S1). Secondary structures of the 5’ UTR of *cyclin B* in *M. galloprovincialis* and *M. californianus* were predicted by inputing 5’ UTR sequences and the start codon (Table S2) into RNAfold (Hofacker 2003). Secondary structures were calculated using default settings and motifs defining thermometers were observed.

## Results

### Heat-stress response gene expression (hsp70)

**Fig. 4:**
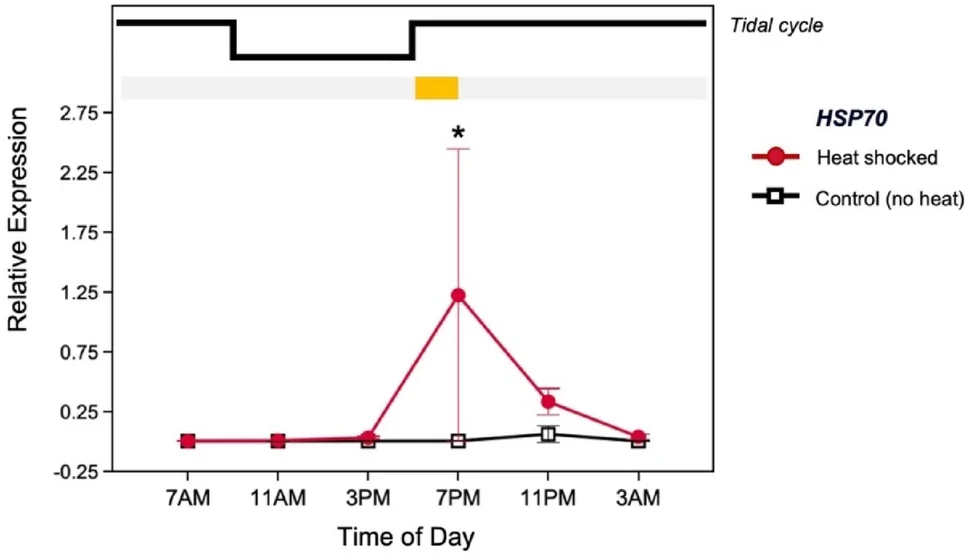
Relative gene expression of *hsp70* (*HSPA12A*) in *M. galloprovincialis* across a 21-h period (6 total time points). Tidal cycle is denoted by the bold black line at the top. Feeding period is denoted by the yellow section below tidal cycle line. Each data point represents the mean of *N* = 25 permuted values from original groups (*N* = 5 mussels sacrificed per group at each time point). Error bars represent ± 2 S.E. from the mean. Asterisks indicate statistical significance (BH-adj. *p* < 0.05) between control/HS group per time point. Temporal analyses are stated within the results section.

To confirm that mussels were appropriately acutely heat shocked, we measured the transcript levels of *hsp70* (*HSPA12A*) (Fig. 4). A one-way ANOVA revealed that *hsp70* relative expression differed significantly between the groups (*F*_11, 288_ = 3.940, *p* < 0.001. *Hsp70* expression in heat-stressed (HS) mussels was significantly elevated at 7PM compared with all other groups (across time points and relative to controls; BH-adj. *p* < 0.01). Notably, expression increased 43-fold at 7PM (high tide) relative to 3PM when HS mussels were experiencing heat stress during low tide (BH-adj. *p* < 0.001). As expected, *hsp70* expression did not vary among time points in control mussels (BH-adj. *p* > 0.05).

### Anti-apoptosis gene expression

**Fig. 5:**
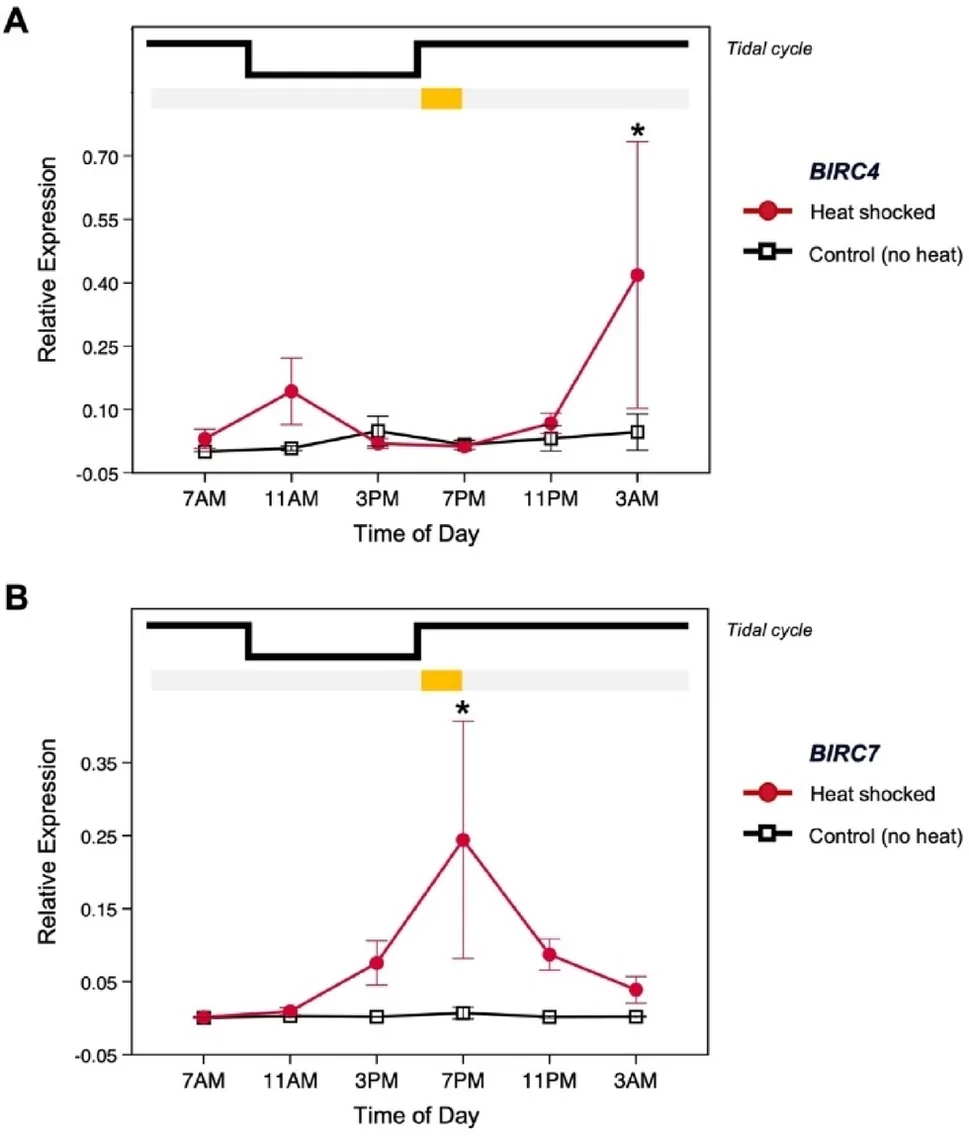
Relative gene expression of anti-apoptosis markers *BIRC4* (**A**) and *BIRC7* (**B**) in *M. galloprovincialis* across a 21-h period (6 total time points). Tidal cycle is denoted by the bold black line at the top. Feeding period is denoted as the yellow section below tidal cycle line. Each data point represents the mean of *N* = 25 permuted values from original groups (*N* = 5 mussels sacrificed per group at each time point). Error bars represent ± 2 S.E. from the mean. Asterisks indicate statistical significance (BH-adj. *p* < 0.05) between control/HS group per time point. Temporal analyses are stated within the results section.

*BIRC4* and *BIRC7* encode caspase-inhibitory proteins that suppress apoptosis and promote cell survival. ANOVA analysis showed significant differences in *BIRC4* expression among treatment groups (*F*_11, 288_ = 5.80, *p* < 0.001; Fig. 5A). In the HS group, *BIRC4* expression was 6.2-fold higher at 3AM relative to the preceding time point (11PM) (BH-adj. *p* < 0.001) and significantly higher than all other time points within the HS group (BH-adj. *p* < 0.001). This 3AM peak expression in the HS group was 9-fold higher than the control group (BH-adj. *p* < 0.001*).* We did not observe any significant variation between time points in the control group (BH-adj. *p* > 0.05).

*BIRC7* expression was significantly different between the experimental groups (*F*_11, 288_ = 8.66, *p* < 0.001; Fig. 5B). At 7PM (re-oxygenation), *BIRC7* expression for the HS group was 3.2-fold upregulated relative to the previous time point (3PM; low tide) (BH-adj. *p* < 0.001). Furthermore, *BIRC7* expression at 7PM was significantly elevated compared to all other time points in the HS group (BH-adj. *p* < 0.001) and 34-fold higher than the 7PM expression in the control group (BH-adj.*p* < 0.001). No significant differences were detected among control group time points across the tidal cycle (BH-adj. *p* > 0.05).

### p53 gene expression

**Fig. 6:**
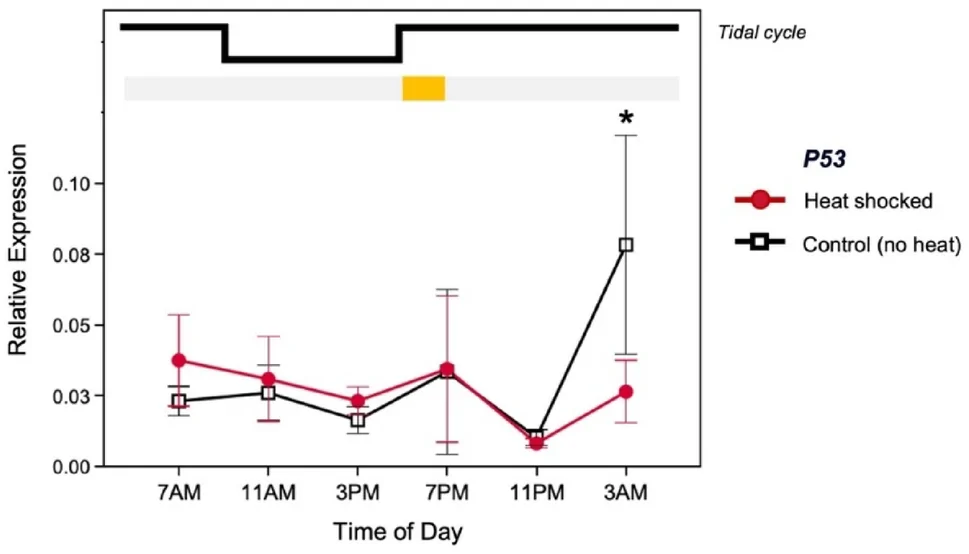
Relative gene expression of *p53* in *M. galloprovincialis* across a 21-h period (6 total time points). Tidal cycle is denoted by the bold black line at the top. Feeding period is denoted as the yellow section below tidal cycle line. Each data point represents the mean of *N* = 25 permuted values from original groups (*N* = 5 mussels sacrificed per group at each time point). Error bars represent ± 2 S.E. from the mean. Asterisks indicate statistical significance (BH-adj. *p* < 0.05) between control/HS group per time point. Temporal analyses are stated within the results section.

We measured *p53* gene expression to investigate cell-cycle arrest at G1/S checkpoint following acute heat stress. One-way ANOVA suggested that the mean of at least one experimental group differed from other groups (*F*_11, 288_ = 4.11, *p* < 0.001; Fig. 6). In the control group, *p53* expression was significantly upregulated at 3AM, with a 7.8-fold increase relative to the preceding time point (BH-adj. *p* < 0.001). At the same time point, *p53* expression was 3-fold higher in the control group compared with the HS group (BH-adj. *p* < 0.001). All comparisons between time points in the HS group were not significant (BH-adj. *p* > 0.05).

### Oxidative stress gene expression

**Fig. 7:**
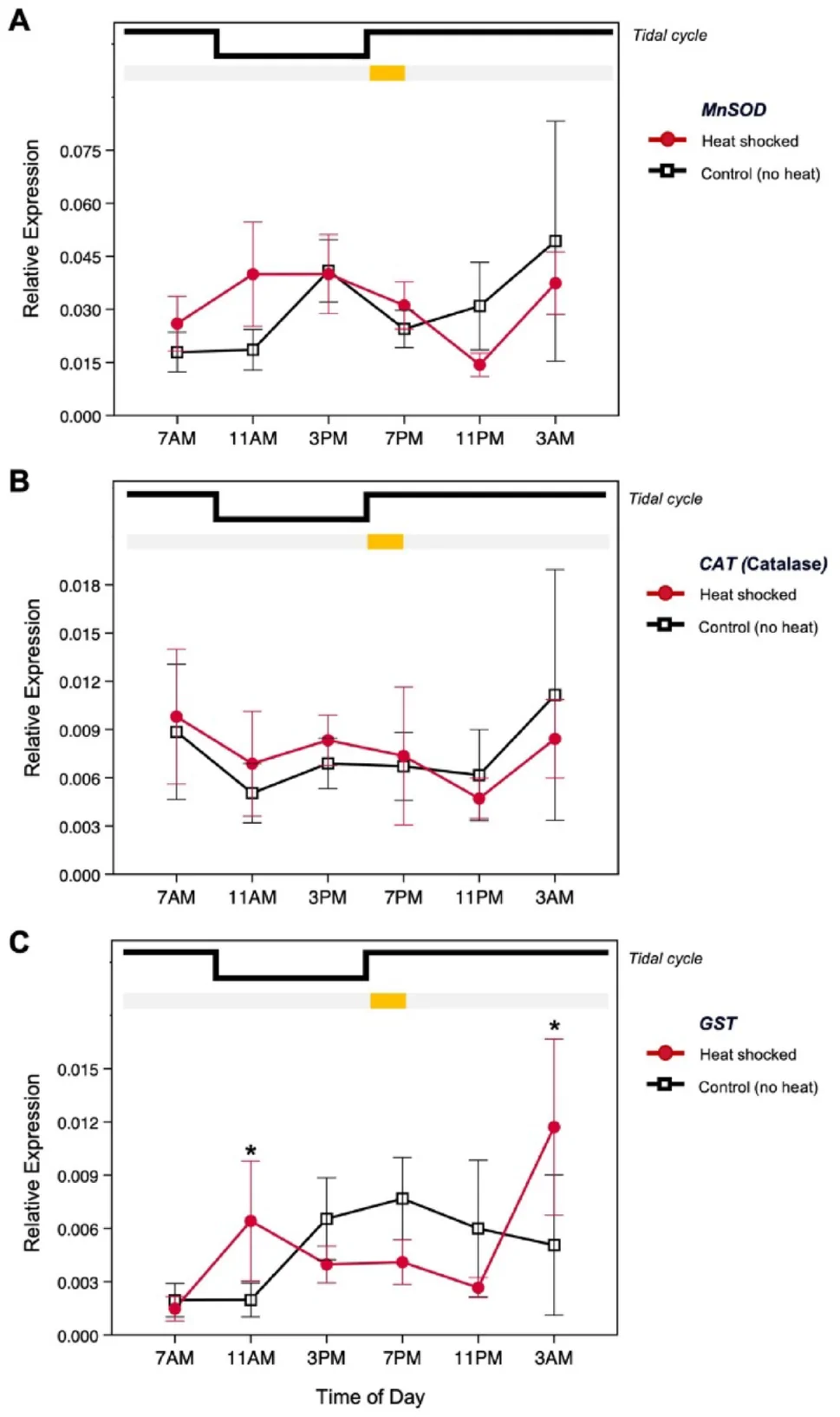
Relative gene expression of oxidative stress markers *MnSOD* (**A**), *CAT* (**B**), and *GST* (**C**) in *M. galloprovincialis* across a 21-h period (6 total time points). Tidal cycle is denoted by the bold black line at the top. Feeding period is denoted as the yellow section below tidal cycle line. Each data point represents the mean of *N* = *25* permuted values from original groups (*N* = 5 mussels sacrificed per group at each time point). Error bars represent ± 2 S.E. from the mean. Asterisks indicate statistical significance (BH-adj. *p* < 0.05) between control/HS group per time point. Temporal analyses are stated within the results section.

To investigate the oxidative stress response in mussels, we measured relative expression of three oxidative stress markers: manganese superoxide dismutase (*MnSOD*), glutathione S-transferase (*GST*), and catalase (*CAT*). ANOVA analysis revealed significant differences in *MnSOD* expression between the experimental groups (*F*_11, 288_ = 2.84, *p* < 0.01; Fig. 7A). Although we did not observe a significant up- or downregulation in *MnSOD* expression in the control mussels between two consecutive time points, control mussels at the beginning of the tidal cycle (7AM; high tide) exhibited significantly lower *MnSOD* expression than control mussels at the later phase of the tidal cycle at 3AM (re-oxygenation; high tide) (BH-adj. *p* < 0.05). No significant variation in *MnSOD* expression was observed between the HS group time points (BH-adj. *p* > 0.05). Additonally, no differences were observed between control and treatment groups.

No significant differences were observed in *CAT* expression between time points for either control or HS group (ANOVA, *F*_11, 288_ = 1.11, *p* > 0.05; Fig. 7B). Furthermore, we did not observe significant differences between the two groups at each time point.

*GST* expression was significantly different between the experimental groups (ANOVA, *F*_11, 288_ = 5.07, *p* < 0.001; Fig. 7C*)*. In the HS group, expression was 4.4-fold upregulated at 11AM (low tide) compared to the preceding time point (7AM; high tide) (BH-adj. *p* < 0.05) and 3.3-fold higher than the control group at the same time point (BH-adj. *p* = 0.053). *GST* expression did not further increase in the subsequent time points for the HS group during low tide. After resubmergence, expression in the HS group was significantly increased by 4.4-fold at 3AM relative to the preceding time point (11PM; BH-adj. *p* < 0.001). Compared to the control group at 3AM, *GST* expression in HS group was 2.3-fold higher (BH-adj. *p* < 0.01). *GST* expression across tides in the control group revealed increased expression during low tide between 11AM and 3PM (BH-adj. *p* = 0.052), and sustained throughout high tide.

### Cyclin D gene expression

**Fig. 8:**
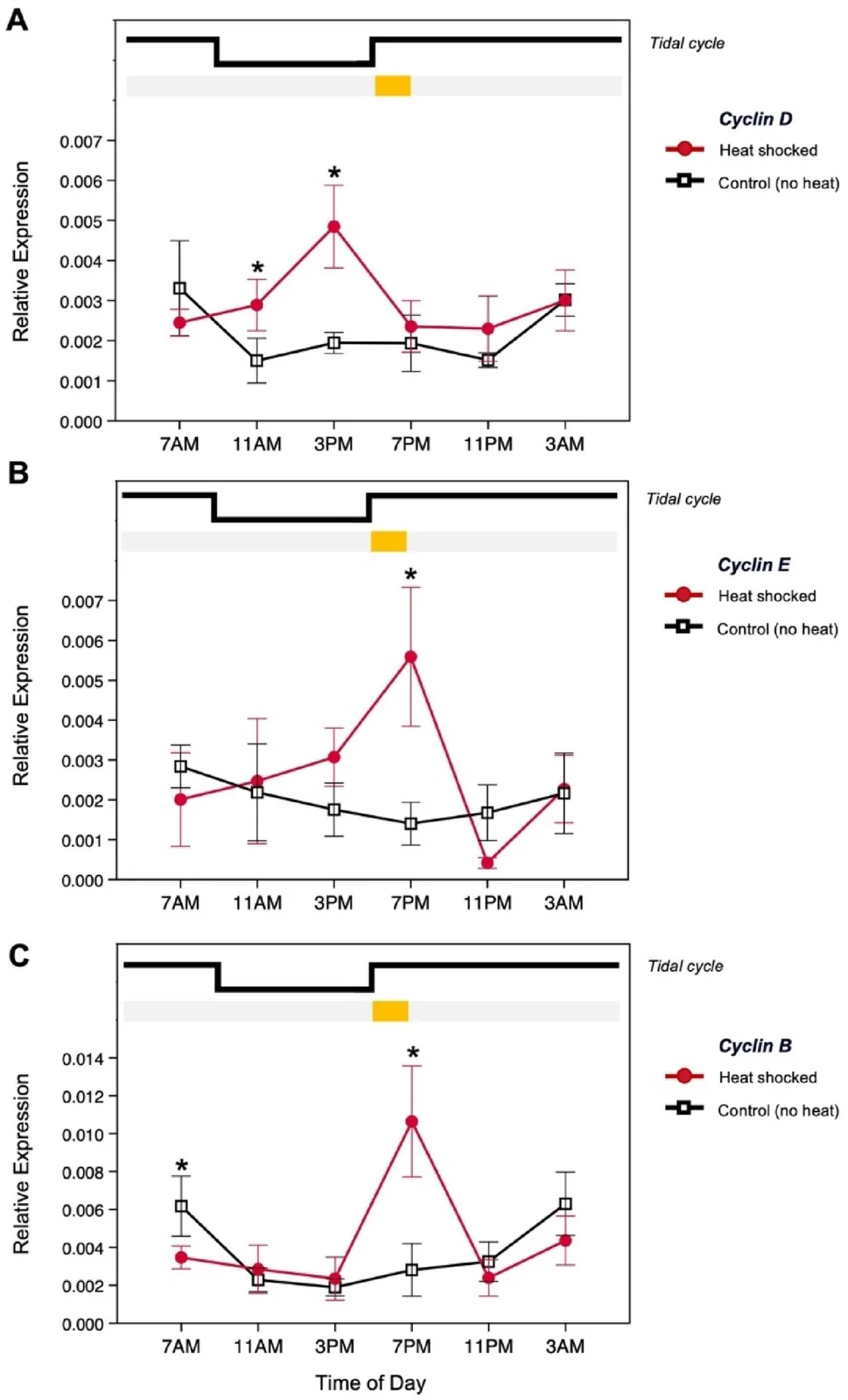
Relative gene expression of cell-cycle regulator markers *cyclin D* (**A**), *cyclin E* (**B**), and *cyclin B* (**C**) in *M. galloprovincialis* across a 21-h period (6 total time points). Tidal cycle is denoted by the bold black line at the top. Feeding period is denoted as the yellow section below tidal cycle line. Each data point represents the mean of *N* = *25* permuted values from original groups (*N* = 5 mussels sacrificed per group at each time point). Error bars represent ± 2 S.E. from the mean. Asterisks indicate statistical significance (BH-adj. *p* < 0.05) between control/HS group per time point. Temporal analyses are stated within the results section.

One-way ANOVA revealed significant differences in mean *cyclin D* expression between experimental groups (*F*_11, 288_ = 7.18, *p* < 0.001; Fig. 8A). In the HS group, *cyclin D* expression peaked at 3PM, increasing 1.7-fold relative to the previous low-tide time point (BH-adj. *p* < 0.001). Additionally, *cyclin D* expression for the HS group at 3PM (low tide) was 2.5-fold higher than the control group at the same time point (BH-adj. *p <* 0.001). In the control group, we observed a 2.2-fold decrease in *cyclin D* expression from 7AM to 11AM (high to low tide) (BH-adj. *p* < 0.01). This lowered expression was maintained until 10 h into high tide, with a 2-fold increase from 11AM to 3AM (BH-adj. *p* < 0.01). The control group expression at 11AM is 1.9-fold lower than the HS group at the same time point (BH-adj. *p* < 0.05).

### Cyclin E gene expression

Significant differences in mean *cyclin E* expression among experimental groups were observed (ANOVA, *F*_11, 288_ = 6.05, *p* < 0.001; Fig. 8B*).* In the HS group, *Cyclin E* expression increased 1.8-fold between low tide and resubmergence (3PM to 7PM) (BH-adj. *p* < 0.01) was 4-fold higher than the control group expression at the same time point (BH-adj. *p* < 0.001). Subsequently, the expression decreased by 14-fold from 7PM to 11PM (BH-adj. *p* < 0.001), before increasing again by 5.4-fold from 11PM to 3AM in the HS group (BH-adj. *p* < 0.05). We did not observe any significant variation in *cyclin E* gene expression between the control-group time points throughout the tidal cycle (BH-adj. *p* > 0.05).

### Cyclin B gene expression

One-way ANOVA revealed significant differences in mean *cyclin B* expression between experimental groups (*F*_11, 288_ = 13.17, *p* < 0.001; Fig. 8C). In the HS group, *cyclin B* expression peaked at 7PM (re-oxygenation) with a 4.5-fold higher expression than the previous time point at 3PM (low tide) (BH-adj. *p* < 0.001). Peak *cyclin B* expression (7PM) in the HS group was 3.8-fold higher than the control group at that time point (BH-adj. *p* < 0.001) and significantly higher than all other time points in both groups (BH-adj. *p* < 0.001). In the control group, *cyclin B* decreased 2.7-fold at from 7AM (high tide) to 11AM (low tide) (BH-adj. *p* < 0.001). This lowered expression was sustained, until it increased by 1.9-fold from 11PM to 3AM during resubmergence (BH-adj. *p* < 0.01). At 7AM, *cyclin B* expression in the control group was 1.8-fold higher than the HS group (BH-adj. *p* < 0.05). *Cyclin B* had the highest peak expression among the three cyclins. It was ~ 2-fold higher than *cyclin D* and *E.*

### RNA/DNA ratio assay

**Fig. 9:**
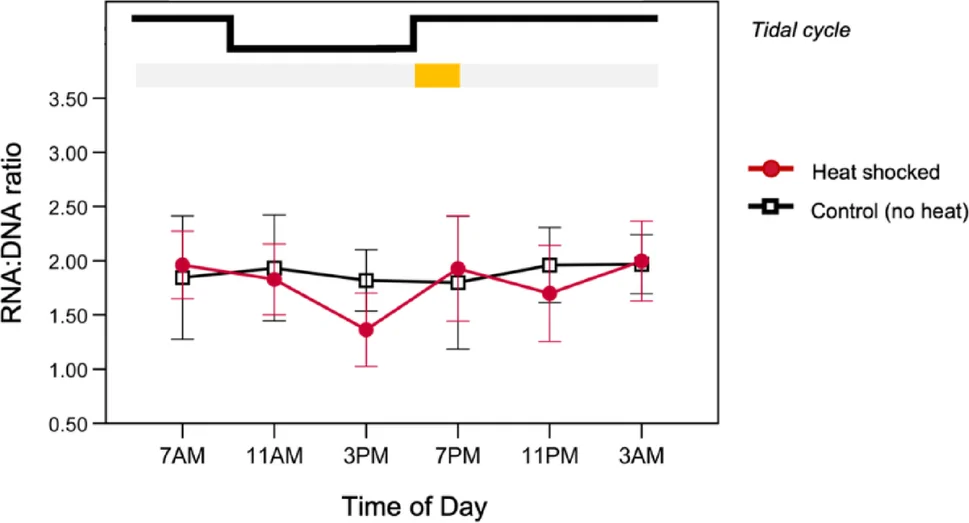
RNA/DNA ratio in M. galloprovincialis across a 21-h period (6 total time points). Tidal cycle is denoted by the bold black line at the top. Feeding period is denoted as the yellow section below tidal cycle line. Values represent mean ± 2 S.E (*N* = *5* mussels sacrificed per group at each time point). No significant difference was observed between the two groups at any time points (one-way ANOVA *p* > 0.05).

To assess whether mussels were exhibiting short-term growth, we measured the concentration of nucleic acids in mussel gill tissues and calculated the RNA/DNA ratio, a method commonly used to indirectly measure growth in marine organisms. Higher ratios are associated with elevated growth rates (Leick 1968; Buckley 1984; Caldarone et al. 2003).We observed no significant differences in RNA/DNA ratio between the control and HS group, nor any fluctuation in growth within each individual group between each timepoint (Fig. 9). The RNA/DNA ratio was ~ 1.5–2 for each group across the entire tidal cycle.

### RNA structure of cyclins D, E, and B

**Fig. 10:**
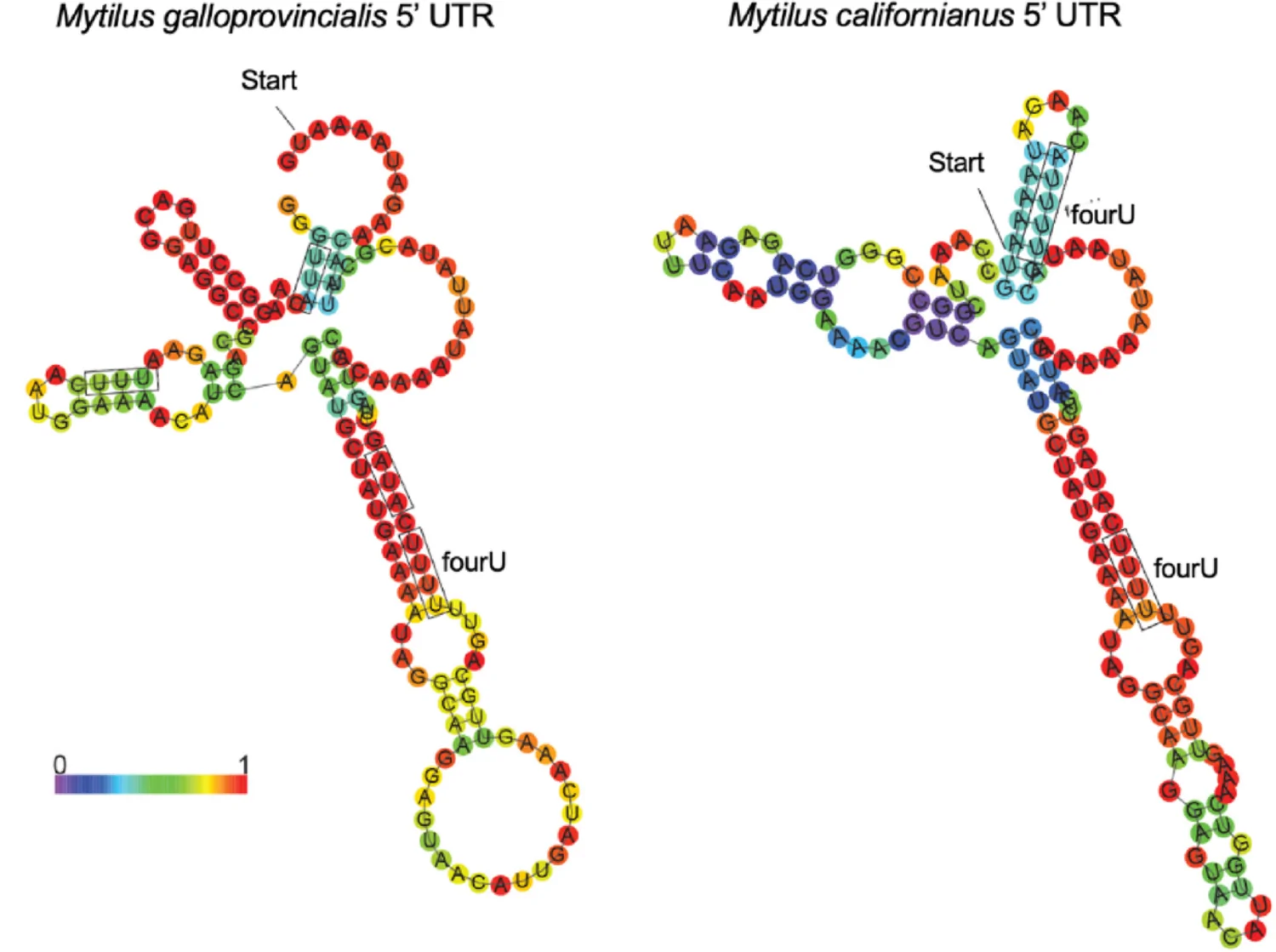
**A** Secondary structure prediction of 5’ UTR of *cyclin B* in *M. galloprovincialis* and **B** *M. californianus.* The structures were generated by RNAfold. U-rich regions boxed, start codon and regions containing four consecutive uracils are labeled. Base-pair probabilities range from 0 (blue) to 1 (red).

We found that the 5’UTR of *cyclin B* is a potential RNA thermometer, but *cyclin D* and *cyclin E* were not likely traditional RNA thermometer candidates. A secondary structure prediction (RNAfold; Hofacker 2003) of the 5’ UTR of *cyclin B* in *M. galloprovincialis* demonstrates a stable structure with a four censecutive uracil (fourU) region sequestered within a hairpin structure, and several A-U base pairs suggesting these elements may confer thermosensitivity (Fig. 10A). For comparison, *M. californianus* shows several A-U base pairs and two fourU motifs (Fig. 10B). Interestingly, one of these motifs contains a run of four consecutive uracils in the terminal hairpin, with a terminal hairpin length typical of RNA thermometers. This arrangement closely resembles the well-characterized and highly prevalent fourU-RNA thermometer motif found in prokaryotes, suggesting a potential thermoregulatory function. FourU-RNA thermometers contain one to four hairpins, with the terminal hairpin having at least four consecutive uracil nucleotides base-pairing with the Shine-Dalgarno sequence (Waldminghaus et al. 2007). The consecutive run uracils in this motif denote temperature sensitivity due to a network of weak hydrogen bonds. Additionally, the start codon (AUG) is sequestered within the terminal hairpin, another characterstic feature observed in some RNA thermometers.

## Discussion

Animals residing in fluctuating environments must coordinate physiological processes by way of temporal partitioning and efficient energy storage. The mammalian cell-cycle model is a temporal cascade of cyclin proteins that when complexed to CDKs phosphorylate target proteins, thereby orchestrating cell division processes throughout the cell . In this study we investigated the gene expression of cyclins in mussels, which make up the temporally variable portion of the protein complex versus CDKs, which are constitutively expressed as observed in mammalian models (Pines 1995; Alberts et al. 2002; Malumbres 2014). We hypothesized that cyclin-mediated cell-cycle progression in mussels is temporally modulated by tidal-regulated oxygen and temperature cycles, consistent with the logic of temporal partitioning in yeast undergoing respiratory cycling (Tu et al. 2005). Under normoxia/hypoxia tidal cycles, we predicted that the cell-cycle cascade would initiate during low-tide functional hypoxia (shell-valve closure) with sequential expression of *cyclin D*,* cyclin E*, and *cyclin B* (mitosis) with peak expression restricted within the low-tide period (Fig. 2). This strategy temporally separates the sensitive processes of the cell cycle (i.e., cell division) from oxidative stress that occurs during high-tide submergence. We further predicted that acute aerial heat-stress during low tide (tide+heat) would delay cyclin progression and shift peak cyclin expression to high-tide resubmergence, as heat combined with functional hypoxia was expected to pose a greater risk of DNA damage than oxidative stress alone (Fig. 2). Finally, we predicted that heat-stressed mussels would exhibit higher overall cyclin transcript abundance relative to the control group, consistent with patterns observed in thermally acclimatized high-shore mussels (Gracey et al. 2008) (Fig. 2).

To investigate the progression of the cell cycle under different environmental regimes, we measured the gene expression of *cyclin D*,* E*, and *B* after acclimation to simulated tides (8 h: 14 h; low tide: high tide). Contrary to our predictions, results revealed that peak gene expression of cyclins in the control mussels (no heat) were not restricted to the low-tide period. *Cyclin D* and *B* significantly varied across the tidal sequence, showing modest peaks (~ 2-fold) during both high-tide periods compared to the corresponding low-tide period that day. Cell division is an energy-dependent set of processes, supporting the notion that cell division processes in mussels are aligned with periods of oxidative respiration and high levels of ATP production during high tide (Salazar-Roa and Malumbres 2017). However, whether the observed variation is directly related to low and high tide or endogenous expression cannot be fully discerned without a subtidal control group. To this point, Connor and Gracey (2011) did not find rhythmic gene expression of *cyclin B * in *M. californianus* after acclimation to simulated isothermic (17 °C) tidal fluctuations (6 h: 6 h; low tide: high tide) using microarray technology. Furthermore, Elowe and Tomanek (2021) did not reveal variable protein expression of *cyclin B* in *M. californianus* acclimated to a similar tidal regime under laboratory conditions. Additionally, we found that *cyclin E* expression did not vary across tidal fluctuations in control mussels, suggesting that mussels may not have been in a state of ‘rapid’ growth during the experiment. To this end, mussel growth rates in small tank systems are lower than those observed in the field (Bayne and Thompson 1970). In this vein, Connor and Gracey (2011) observed higher gene expression of *cyclin B* in *M. californianus* in nature compared to the laboratory conditions. It is important to note that cell-cycle processes at the protein abundance level have been shown to progress in low oxygen environments in distantly related hypoxia tolerant organisms including shrimp and crayfish (Nuñez-Hernandez, 2018; Singh and Story, 2022). However, bivalves serve as more appropriate models for advancing knowledge of the underlying architecture of cell-cycle dynamics in facultative anaerobic animals enduring cyclical environments. To this end, there is a severe lack of studies exploring cell-cycle dynamics in mussels within intertidal regimes.

In contrast to the control mussel group, acutely heat-stressed mussels at 25 °C demonstrated an alternative pattern of gene expression. Transcript abundance of cyclins in heat-treated mussels was similar to control mussels during the early submergence period. However, this expression significantly differed afterward. Peak expression of cyclins in the heat-stressed mussels was significantly higher compared with the control group, supporting our prediction that heat-stressed mussels would exhibit higher overall cyclin transcript abundance relative to the control group. There were 2–4-fold differences in peak RNA-abundance between control and treatment groups amongst cyclins, with *cyclin B* displaying the highest overall expression. However, these results did not support our inference that low-tide heat stress would delay cyclin expression to cooler environments associated with submergence. We observed peak expression of *cyclin D* during low-tide heat followed by *cyclin E* and *B* during the re-oxygenation period at 7PM. The mammalian cell-cycle model reveals that protein expression of *cyclin D* precedes *cyclin E* and *B* as the cells transition between growth, DNA synthesis, and ultimately cell division (Vermeulen et al. 2003b). Therefore, our gene-expression results in the acute group align with this orderly nature of cyclin protein expression events. Additionally, the mammalian cell cycle spans 24 h, consisting of a 23-h interphase (G_1_, S and G_2_ phases), while the mitosis phase only lasts for ~ 1 h (Yang 2018). However, we only observed an 8-h interval between the peak of *cyclin D* and *cyclin E*,* B* transcripts suggestive of species-level differences in cell-cycle programs.

Our findings potentially align with recent evidence that stress can induce cell-cycle synchronization, such that cell-cycle regulators, checkpoints, and progression occur in a staggered and partially overlapping manner, rather than progressing in strict, phase-by-phase sequence of cell-cycle events (Boye and Nordström 2003; Chen et al. 2012; Bektik et al. 2018). Ultimately, these observations suggest that heat stress or the combination of acute heat stress and functional hypoxia modulated gene expression in mussels. Consistent with our findings, Gracey et al. (2008) reported elevated expression of cell division genes in high-shore mussels (*M. californianus*) exposed to heat shock compared with low-shore mussels experiencing cooler conditions. Importantly, *cyclin B* (mitosis) was the most highly expressed gene within the cell division cluster of analyzed genes. Similarly, Connor and Gracey (2011) reported rhythmic *cyclin B* expression in intertidal mussels in the field, whereas fully submerged individuals exhibited arrhythmic expression, supporting the inference of environmental modulation of *cyclin B* gene expression. In contrast, Li (2022) revealed that extreme laboratory-induced stress (7 days; air+heat) suppressed gene expression of cell division genes in the intertidal oyster *C. virginica*. Although mussels grow more slowly under warm laboratory conditions given that all other variables are held constant (Fitzgerald-Dehoog^2012^), elevated cyclin expression under moderate heat stress in air may prime cells for future growth rather than directly reflecting active growth rate (e.g., tissue accretion).

We proposed that the higher expression of cyclins in the heat-stressed mussels may be related to mitigation of higher cell turnover or thermodynamics. RNA thermometers provide a level of thermal regulation of protien abundance, revealed in unicellular models by preventing translation until a certain temperature threshold is reached (Sharts et al. 2024). They are typically structured noncoding RNA sequences located in the 5′ UTRs of genes that contain temperature-sensitive motifs due to a network of weak hydrogen bonds in their secondary structure (Sharts et al. 2024) To this end, secondary RNA structures may have been selected for in intertidal organisms. Here, we analyzed the possibility of secondary structure regulation in the 5’ UTRs of *cyclins D*, *E*, and *B*. Remarkably, we found that *cyclin B* RNA possessed classical structures in *M. galloprovincialis* and closely related *M. californianus* (Distel 2000), suggesting that its translation is thermally regulated, but not *cyclin D* and *E*. The effect of RNA thermometers on gene expression has not been analyzed in mussels. However, it is important to note that *cyclin B* as a thermometer candidate, had roughly two-times greater RNA abundance than *cyclin D* and *E*. Importantly, Gracey et al. (2008) revealed robust oscillations of *cyclin B* (EST in Fig. 10) in thermally stressful field conditions. In this vein, work by Pines and Hunter (1990) revealed higher levels of gene expression in *cyclin B* versus *cyclin A* in cultured human cells and suggested that the difference was a result of higher stability of *cyclin B* RNA through protein-binding within its 3’ UTR. With regard to the functionality of 5’ UTR’s, Wan et al. (2012) revealed positive correlations between 5’ structure complexity, RNA stability, and temperature across the yeast transcriptome. Furthermore, they observed that RNA thermometors avoided exome mediated degradation following heat stress as a testament to their thermal stability and providing an explanation for higher transcript abundance under heat stress. Hence, nature may have selected for a complex axis of gene expression, UTR structure, protein binding, and temperature that modulates cell-cycle dynamics in mussels. Further work to validate and characterize the 5’ UTR of *cyclin B* as an RNA thermometer across different temperatures is intriguing, requiring translation-dependent gene-expression assays and structural characterization under different temperatures. Currently, our gene-expression and RNA thermometer analyses provide further insight of the architecture of cell cycle in mussels.

To further determine whether the upregulation of cyclin gene expression resulted in short-term organismal growth, we performed an RNA/DNA ratio assay. Previous studies showed a positive correlation between RNA/DNA ratio with growth rate in bivalves (Blicher et al. 2010, Yeung and Leung 2013; Wang et al. 2017). Our results showed that despite the upregulation of cyclin gene expression in the heat-stressed group, RNA/DNA ratios indicated no detectable short-term growth. Although an RNA/DNA ratio greater than 1 is associated with increased RNA synthesis necessary for growth, it is unlikely that growth was promoted within the short observation time. The short duration of acute heat shock exposure may have only cued preparatory processes for cell division and growth. Energetic conditions may not have been ideal (e.g., glycogen storage) and growth was halted (Busti et al. 2010; Yuan et al. 2013; Lee and Finkel 2013). Similar to our findings, Singh and Story (2022) found higher co-expression of cyclin and CDK inhibitor proteins in growing crayfish under hypoxia compared to individuals acclimated to normoxic conditions. Lastly, shell growth positively coincides with tissue growth highlighting the need for microscopy analysis of shell accretion within cell-cycle studies.

### Heat-stress and anti-apoptosis responses

Inducible heat shock protein response in mussels and bivalves is well observed in subjects exposed to acute heat shock or acclimated/acclimatized to cyclic shock in laboratory and field settings (Roberts et al. 1997; Franzellitti and Fabbri 2005; Tomanek and Zuzow 2010). Observations of the oyster genome revealed eighty-eight heat shock 70 genes revealing its importance for survival in the intertidal zone (Zhang et al. 2012). *HSPA12A* is a member of the hsp70 gene family and manages the refolding of denatured proteins. In its role as a protein chaperone, mussels in the heated regime revealed robust expression of *HSPA12A*. The maximum temperature reached in the tank was 25 °C which is above the reported heat-shock induction temperature of at least one hsp70 gene within *M. galloprovincialis* (Georgoulis et al. 2021). Connor and Gracey (2011) revealed that the orthologous counterpart in *M. californianus* is transiently expressed rhythmically during cool low tides as well as large inductions after acute heat stress. Therefore, *HSPA12A* may also be a component of anticipatory mechanisms in some bivalve species. Lastly, Connor and Gracey (2020) revealed a series of alternative heat-shock genes that are expressed during the heat-stress period in heat acclimated mussels. Similarly, Tomanek and Zuzow (2010) revealed the expression of a suite of heat shock proteins in *M. galloprovincialis* following acute heat shock.

Inhibitors of apoptosis (IAP) genes *BIRC4* and *BIRC7* play a role in inhibiting programmed cell death by suppressing caspase activation and promoting cell survival under stressful conditions (Dubrez-Daloz et al. 2008). These genes were significantly elevated in the heat-stressed group during the resubmergence period. *BIRC* genes have been observed to respond to heat stress across several bivalve species (Connor and Gracey 2011; Lim et al. 2016; Song et al. 2021; Wang et al. 2023). The elevated expression of *BIRC4* and *BIRC7* following moderate heat stress may reflect a cellular strategy to avoid apoptosis and instead promote survival and stress tolerance during recovery. Work on cancer cells suggests that apoptosis occurs under extreme stress, wheras senescence (cell-cycle arrest at the G_1_/S checkpoint) occurs following mild stress (Campisi 2013; Gewirtz 2013; Wiley and Campisi 2016). The tumor suppressor p53 induces cellular senescence in response to stress-induced DNA damage (Levine 1997; Vousden and Prives 2009). In our study, *p53* expression was only moderately upregulated approximately 10 h after resubmergence in control mussels. Notably, *p53* was not differentially expressed in the HS group. The lack of *p53* expression in heat-stressed mussels indicates that DNA damage may have been prevented by thermally induced chaperones in their anti-apoptotic role and mediator of cell fate (Sottile and Nadin 2018). Importantly, p53 induces senescence by decreasing intracellular levels of *cyclin B* (Innocente et al. 1999). Hence, the lack of *p53* gene upregulation in mussels after heat-stress may be related to the elevated levels of *cyclin B* transcript abundance and sustained cell viability. A study by Han et al. (2013) revealed low gene expression but high protein abundance of p53 in heat-stressed human cells suggesting that post-transcriptional processes were more important than transcript abundance in mediating heat-stress responses (Han et al. 2013). Relatedly, Li (2022) found low gene expression of *p53* and elevated expression of IAPs in oyster *C virginica* subjected to extreme multistress (7 days; air+heat). Further investigations into the biochemistry of p53 are required to unveil its functions in mussels (Balvan et al. 2015).

### Oxidative stress response

We predicted that the cell-cycle cascade in control mussels would occur during low tide to minimize oxidative stress associated with resubmergence and reinitiation of aerobic metabolism. To assess oxidative-stress response, we measured the relative expression of three oxidative-stress markers: manganese superoxide dismutase (*MnSOD*), glutathione S-transferase (*GST*), and catalase (*CAT*). Oxidative-stress risk increases during re-oxygenation following low tide due to elevated respiration and reactive oxygen species (ROS) production during oxygen debt mitigation. This risk is further exacerbated by heat stress in marine bivalves (Liu et al. 2018; Khan et al. 2021; Xu et al. 2022). An increase in antioxidant enzyme activity following periods of hypoxia has been observed in many species (Lushchak et al. 2001).

MnSOD converts harmful ROS into less damaging forms, and catalase further breaks them down, thus preventing the accumulation of these potentially harmful reactive species (Weydert and Cullen 2010; Liu et al. 2022). We did not observe significant variation in *CAT* expression and minimal differences in *MnSOD* expression. Furthermore, we did not observe *MnSOD* upregulation in heat-stressed mussels throughout the tidal cycle. Alternatively, *GST* expression in the heat-stressed group was significantly upregulated during early low tide. Previous studies indicate that intertidal invertebrates upregulate endogenous antioxidants during low tide as an anticipatory strategy to combat ROS upon resubmergence (Hermes-Lima and Zentero-Savin 2002; Welker et al. 2012; Giraud-Billoud et al. 2019). Thus, *GST* induction during low tide in the heat-stressed group may reflect an anticipatory response to tidally driven functional hypoxia. Furthermore, the significant *GST* upregulation at 3AM suggests a delayed response to the increased oxidative stress upon reentry into the water, more pronounced than in controls and consistent with published findings. Lastly, the lack of *CAT* and *MnSOD* induction suggests that oxidative stress was possibly mitigated through modest *GST* upregulation in the control group. Furthermore, the lack of induction of *CAT* and *MnSOD* under heat stress could be a result of heat shock proteins (i.e., hsp70 family) providing multifaceted cellular protection, including defense against oxidative stress through the reduction of ROS levels (Kregel 2002; Murshid et al. 2013; Nasr et al. 2019). Therefore, the upregulation of *hsp70* observed in the heat-stressed group during resubmergence may have alleviated oxidative stress, thereby diminishing the need for elevated *CAT* and *MnSOD* expression.

## Conclusion

Our findings support previous observations that environmental stressors (e.g., heat stress and/or hypoxia) can influence the expression of cell cycle-related genes in mussels. Despite our observations of elevated transcript abundance of cyclins in the heat-stressed mussels, we did not detect corresponding short-term differences in RNA/DNA ratios, suggesting that changes in gene expression were not accompanied by measurable changes in growth over the experimental period. This aligns with our existing knowledge that mussel growth is indeterminate and sensitive to the surrounding environment which can promote or hinder tissue or shell accretion. Because we were unable to distinguish the effects of isolated heat stress from those of combined heat and hypoxia exposure, future studies are needed to evaluate potential interactive effects of these stressors. Additional factors known to influence growth and cell-cycle regulation, including food availability, temperature, and exposure duration, also warrant further investigation (Salazar-Roa 2017).

A limitation to our study is that our observations were heavily based upon gene-expression data, which may not align with protein expression or activity, particularly given the limited availability of commercial antibodies for protein analyses in non-model organisms. However, proteomic studies of bivalves continue to emerge (Tomanek 2014). Overall, these findings provide valuable insight into molecular responses to environmental stress in mussels and contribute to our understanding of how facultative anaerobes respond to fluctuating environmental conditions. Furthermore, our analyses of 5’ UTRs (RNA thermometors) and their possible effects on gene expression expands our vision of how eukaryotes cope with thermal stress (Somero 2018).

## Supplementary Information

Below is the link to the electronic supplementary material.

Supplementary Material 1
