# Supplementary material for: Cell-cycle genes in the mussel Mytilus galloprovincialis are cued by aerial heat stress in a fluctuating tidal environment
Source: J Comp Physiol B. 2026 Sep 9;196(5):651–66. doi: 10.1007/s00360-026-01691-y (PMC13658524; doi:10.1007/s00360-026-01691-y)
Supplement: Supplementary file 1 [file 360_2026_1691_MOESM1_ESM.docx]

**Table S1*.*** *Mytilus galloprovincialis* gene primer pairs and accession numbers for RT-qPCR analysis. All accession numbers were retrieved using BLAST (NCBI) and represent transcriptome shotgun assembly (TSA) sequences.

| **Gene name (symbol)** | **Function** | **Forward** | **Accession no. (NCBI)** |
| --- | --- | --- | --- |
| Tubulin A (*TUBA1A*)  **REFERENCE GENE** | Major constituent of microtubules | Forward: CTTCGGTGGTGGTACTGGAT  Reverse: AGTGCTCAAGGGTGGTATGG | GHIK01112497.1 |
| Heat shock protein 70 - 12kDA (*HSPA12A*) | Protein quality control, stress protection (recovery period) | Forward: TACCTGGTCGTTGGCTATG  Reverse: CGACGATTGAGAGGGCAAG | GAEM01001309.1 |
| Baculoviral IAP repeat-containing protein 4 (*BIRC4*) | X-Linked Inhibitor of Apoptosis | Forward: CGGTGCCCACAGTTAACAAA  Reverse: TCAACAGTCCATTTGCAGCA | GHIK01078977.1 |
| Baculoviral IAP repeat-containing protein 7 (BIRC7) | X-Linked Inhibitor of Apoptosis | Forward: ACTGGGTGAATGCTTGTGTTC  Reverse: TTGCCTTGGACAGAACATGC | GHIK01197318.1 |
| G2/mitotic-  specific cyclin-B1 (*CCNB1*) | G2/M-specific cyclin B | Forward: AGCAGCAGCAGCATTACAGA  Reverse: ATTTGGGTCACACATCGACA | GGUW01030666.1 |
| G1/S-specific cyclin-D2 (*CCND2*) | G1/S-specific cyclin D2 | Forward: ATTGGGAAACCTGCATCAAA  Reverse: GTGTTGTTGGTTGGCCTTCT | GHIK01068206.1 |
| G1/S-specific cyclin-E  (*CCNE1*) | G1/S-specific cyclin E | Forward:  TGTGCCAAGGTCAGGGTTAA  Reverse:  TCTCCAACCCTTCCAACACA | GGUW01036564.1 |
| Tumor suppressor protein p53 (*TP53*) | Promotes cell cycle arrest to allow DNA repair and/or apoptosis | Forward: GCCCAAATCATGCCACATCT  Reverse: ATTCTGAGCCAGCTTGAGGT | GHIK01192182.1 |
| Manganese superoxide dismutase MnSOD (*SOD2*) | Encodes an antioxidant enzyme that protect cells from oxidative damage | Forward: GATGCAGCAGTAGCAGTCCA  Reverse:  GTAGGCATGCTCCCAGACAT | JN863295.1  (Source: Wang et al., 2013) |
| Catalase (*CAT*) | Encodes an antioxidant enzyme that protect cells from oxidative damage | Forward:  CTCTGACCGTGGAACCCCTGA  Reverse:  ATCACGGATGGCATAATCTGGA | AY743716.2  (Source: Giannetto et al., 2017) |
| Glutathione S-transferase (*GST*) | Encodes an antioxidant enzyme that protect cells from oxidative damage | Forward: AACTGACCACTTCAAGAATATGCC  Reverse:  AGAAAGTCTGCCATTTACAAAGCT | AF527010.1  (Source: Boukadida et al. 2017) |

Wang, B., Li, L., Chi, C. F., Ma, J. H., Luo, H. Y., & Xu, Y. F. (2013). Purification and characterisation of a novel antioxidant peptide derived from blue mussel (Mytilus edulis) protein hydrolysate. Food chemistry, 138(2-3), 1713-1719.

Giannetto, A., Maisano, M., Cappello, T., Oliva, S., Parrino, V., Natalotto, A., De Marco, G. and Fasulo, S., 2017. Effects of oxygen availability on oxidative stress biomarkers in the Mediterranean mussel Mytilus galloprovincialis. Marine Biotechnology, 19(6), pp.614-626.

Boukadida, K., Cachot, J., Clérandeaux, C., Gourves, P. Y., & Banni, M. (2017). Early and efficient induction of antioxidant defense system in Mytilus galloprovincialis embryos exposed to metals and heat stress. Ecotoxicology and environmental safety, 138, 105-112.

**Table S2**. 5’ UTR sequences for RNAfold analysis

| **Gene** | **5’ UTR** | **Accession no.** |
| --- | --- | --- |
| *Cyclin D* | ATTAAACCATACACGAGGGAAATGGTTGCAGAATGGATGTTCGAGGTATCAATTTCACGCATGCATATATGAGTGACAAGTGCTACATAGTTGAGTAAATGAATAGGATTTCACAGTGTGACCGGGGGTGGTATATAATCACAGCTCCCTAGACTATCAGATTGTTGGCCGTTGAGCACCTTGGAGCATATAAGAGAGAAGGACACACCAACTCATTCATTCATTGTTGGAAGTTCAAAGATAACAGTCAGTGCAGCATTCGGCCAAACGAAAGTCATATAAATACATTGTGTCACTTGTTCATTTCATCCGGAGGATATTATTTATTTACTTATTTATTTTCTGTGCGTTTGTGTTCGATATCATTATTAATTGTAACTTACAAAATTTATTTGCAATAATAATGGATTTGATGTGTTGTGAGGCTGAATCTTTAAAAAGAGCGTACGAAGATCCAGTACTGTTAAAAGATGACAGAGTTTTGCACAATTTACTTGCTACAGAAGACAAATATCAGCCATCAGCAAGTTATTTTAAATGTGTTCAATCAGACATTAAACCATACATGAGGAAAATGGTTGCAGAATGGATG | GHIK01068206.1 |
| *Cyclin E* | GAACGTCGAAGGATGAACACGGGTATTGCAATGGATTAACAAAAAATAGAGGAAATAGCCAATGAATGTTATAATTACAGCAGAGAAGGATAAAGGAATCTGCGACGAGAATG | GGUW01036564.1 |
| *Cyclin B*  (*M. galloprovincialis*) | GGGTTACAAGGCCTTGACGGAGGCCGCAGAGAATTTCAATGGAAAACATCAGTATGCTATGAAAATAGGCAAGGAGTAACATTGATCAAAGTTGCAGTTTTTTCATAGCTAGTGCACAAAATATTATACGCTTAACAAGATAAAATG | GGUW01030666.1 |
| *Cyclin B*  (*M. californianus*) | CCAAAUCGGCCGGGUCAGAGAAUUUCAAUGGAAAACGUCAGUAUGCUAUGAAAAUAGGCAAGGAGUAACAUUGGUCAAAGUUGCAGUUUUUUCAUAGCUGAUGCAAAAAAUAUAAUACGUUUUACAAGAUAAAAUG | ES738621.1 |

**Figure S1**. IGV plot of Cyclin B-ES738621.1(**A**) and Cyclin E (**B**) in *Mytilus californianus*. From top to bottom we show the chromosome and a red vertical line signifying the location of the window for each panel, followed by the kb window position with Cyclin B starting in the 53.5 kb region of the chromosome and Cyclin E in the 68.4 kb region of its respective chromosomes, NW_026262679 and NW_026262701, denoted by a horizontal black line. This is followed by the track for the transcript from the GTF annotation of the *M. californianus* genome in pastel green, followed by the blast results of the swissprot Cyclin B gene in pastel blue. The track below that is for the original 45k EST’s including (ES738621.1) that aligned to that region of the genome in red, followed by the UTR annotation in gold, and finally the start codon for each gene in green.

**Figure S1**. IGV plot of Cyclin B-GGUW01030666.1 (**A**), Cyclin E-GGUW01036564.1 (**B**), and Cyclin D2- GHIK01068206.1(**C**) in *Mytilus galloprovincialis*. From top to bottom we have the same as configuration as **above**, starting with the chromosome (black and red vertical line), followed by the window position (black number line) for chromosomes NC134841, NC134843 and NC134847, the transcript (blue) from *M. galloprovincialis*, ESTs (red), UTRs (gold) and start codon (green).

**Figure S2.** Secondary structure prediction of **A)** clycin D2 5’ UTR and **B)** cyclin E 5’ UTR in *M. galloprovincialis.* Structures generated by RNAfold. Both structures lack a terminal hairpin containing a run of uracils or any known RNA thermometer motifs.

**Figure S3**. Summary of the relative expression levels of all genes. Only genes exhibiting peak expression within each group are labeled on the graph. The indicated peaks are arbitrary and are shown for illustrative purposes only; they do not represent actual experimental data.
